# Moyamoya Disease in a Patient With Cerebral Palsy Presenting With Intraventricular Hemorrhage and Hydrocephalus Requiring Ventriculoperitoneal Shunt Placement: A Case Report

**DOI:** 10.7759/cureus.83804

**Published:** 2025-05-09

**Authors:** Christian P Howard, Kevin Szafran, Jake Lester, Brandt Gruizinga

**Affiliations:** 1 Medicine, Nassau University Medical Center, East Meadow, USA; 2 General Surgery, Nassau University Medical Center, East Meadow, USA

**Keywords:** acute hemorrhagic stroke, cerebral palsy (cp), intraventricular hemorrhage (ivh), moya moya disease, moya moya syndrome, post-hemorrhagic hydrocephalus, ventriculoperitoneal (vp) shunt

## Abstract

Moyamoya disease (MMD) is a chronic cerebrovascular disorder characterized by progressive stenosis of the internal carotid arteries (ICA) and the development of fragile collateral vessels. These abnormal vessels can lead to ischemic or hemorrhagic strokes, with intracranial hemorrhage being a notable complication. Although rare, hydrocephalus may also occur in MMD, particularly in the setting of intraventricular hemorrhage (IVH). We present the case of a 42-year-old male with a history of cerebral palsy (CP) and MMD, diagnosed at the age of seven, who developed IVH and hydrocephalus. Despite prior bilateral craniotomy and encephaloduroarteriosynangiosis (EDAS), the patient presented with seizure activity and was found to have a hemorrhagic stroke with IVH and hydrocephalus on imaging. He initially underwent external ventricular drain (EVD) placement, which was ineffective, necessitating ventriculoperitoneal (VP) shunt insertion. His hospital course was complicated by aspiration pneumonia and chronic respiratory failure, requiring tracheostomy. Although the IVH and hydrocephalus ultimately resolved, the patient remained in a vegetative state. This report highlights the complexities of managing MMD in patients with comorbid CP, underscoring the need for early diagnosis, timely revascularization, and coordinated multidisciplinary care.

## Introduction

Moyamoya disease (MMD) is a chronic cerebrovascular disorder characterized by progressive stenosis of the internal carotid arteries (ICA) and their major branches, leading to the development of fragile collateral vessels. The term moyamoya, meaning "hazy puff of smoke" in Japanese, describes the characteristic angiographic appearance of these abnormal collaterals. MMD can present with ischemic or hemorrhagic strokes, seizures, and cognitive impairment. In adults, intracranial hemorrhage is more common due to the rupture of fragile collateral vessels [1].

The formation of these collateral vessels in MMD results from chronic hypoperfusion caused by stenosis of the ICA and its branches. Hypoxic stress stimulates vascular endothelial growth factor (VEGF), promoting compensatory angiogenesis from other arteries. However, these newly formed vessels are thin-walled, tortuous, and weak, predisposing patients to hemorrhagic strokes, endothelial damage, and thrombosis, which can lead to embolic ischemia [2]. Hydrocephalus is a rare complication of MMD, with one large review study estimating an overall incidence of 0.7%, and post-hemorrhagic hydrocephalus is even rarer [3]. 

A strong genetic component has been identified in MMD, particularly in East Asian populations. The RNF213 mutation (R4810K variant) on chromosome 17q25.3 is the most well-established risk factor, significantly increasing disease susceptibility. Other implicated genes, including ACTA2, GUCY1A3, PTPRN2, and BRCC3, contribute to vascular dysfunction, arterial remodeling, and impaired angiogenesis. Familial cases often follow an autosomal dominant inheritance pattern with incomplete penetrance, suggesting an interplay between genetic predisposition and environmental factors [4].

Moyamoya syndrome (MMS) refers to a similar clinical presentation; however, it occurs secondary to underlying conditions such as Down syndrome, neurofibromatosis type 1, sickle cell disease, prior cranial irradiation, autoimmune disorders, and atherosclerosis, among others [5]. Diagnosis relies on cerebral angiography, the gold standard for detecting stenosis and collateral vessel formation. Additional imaging modalities, including MRI, MRA, CT, and perfusion studies, provide supportive evidence [6]. Treatment focuses on stroke prevention, with surgical revascularization procedures such as superficial temporal artery-to-middle cerebral artery (STA-MCA) bypass and encephaloduroarteriosynangiosis (EDAS) being the most effective strategies for improving cerebral perfusion [7]. In patients who develop hydrocephalus, management includes external ventricular drains (EVD) and ventriculoperitoneal (VP) shunt placement. Despite intervention, disease progression remains a concern, necessitating long-term monitoring.

We present a rare case of a patient with MMD and cerebral palsy (CP) who developed intraventricular hemorrhage (IVH) and hydrocephalus, requiring EVD placement followed by VP shunt insertion and subsequent revision. This report sheds light on the challenges in managing MMD in patients with comorbid CP and highlights the importance of early diagnosis, timely revascularization, and a multidisciplinary approach to optimize patient outcomes.

## Case presentation

Patient information

A 42-year-old Asian male born with CP and a past medical history of hypertension, gastroesophageal reflux disease (GERD), and coronary artery disease (CAD) presented with new-onset seizure activity. He had been diagnosed with MMD at the age of seven following a left-sided ischemic stroke, and subsequently undergone bilateral craniotomy with EDAS. He had residual left-sided weakness, aphasia, and dysphagia, and communicated using an iPad due to non-verbal baseline status. He had a remote history of seizures but was not on any anti-seizure medications. He had no known recurrent strokes until this presentation.

Clinical presentation

The patient had been found seizing at his group home, exhibiting right-sided twitching and horizontal nystagmus. On arrival at the emergency department, he was postictal, non-verbal, with a Glasgow Coma Scale (GCS) score of 8. He was intubated for airway protection due to emesis and decreased responsiveness. Vital signs were notable for hypotension (BP: 98/64 mmHg), bradycardia (HR: 65 bpm), and oxygen saturation of 98% on supplemental oxygen.

Hospital course

**Figure 1:**
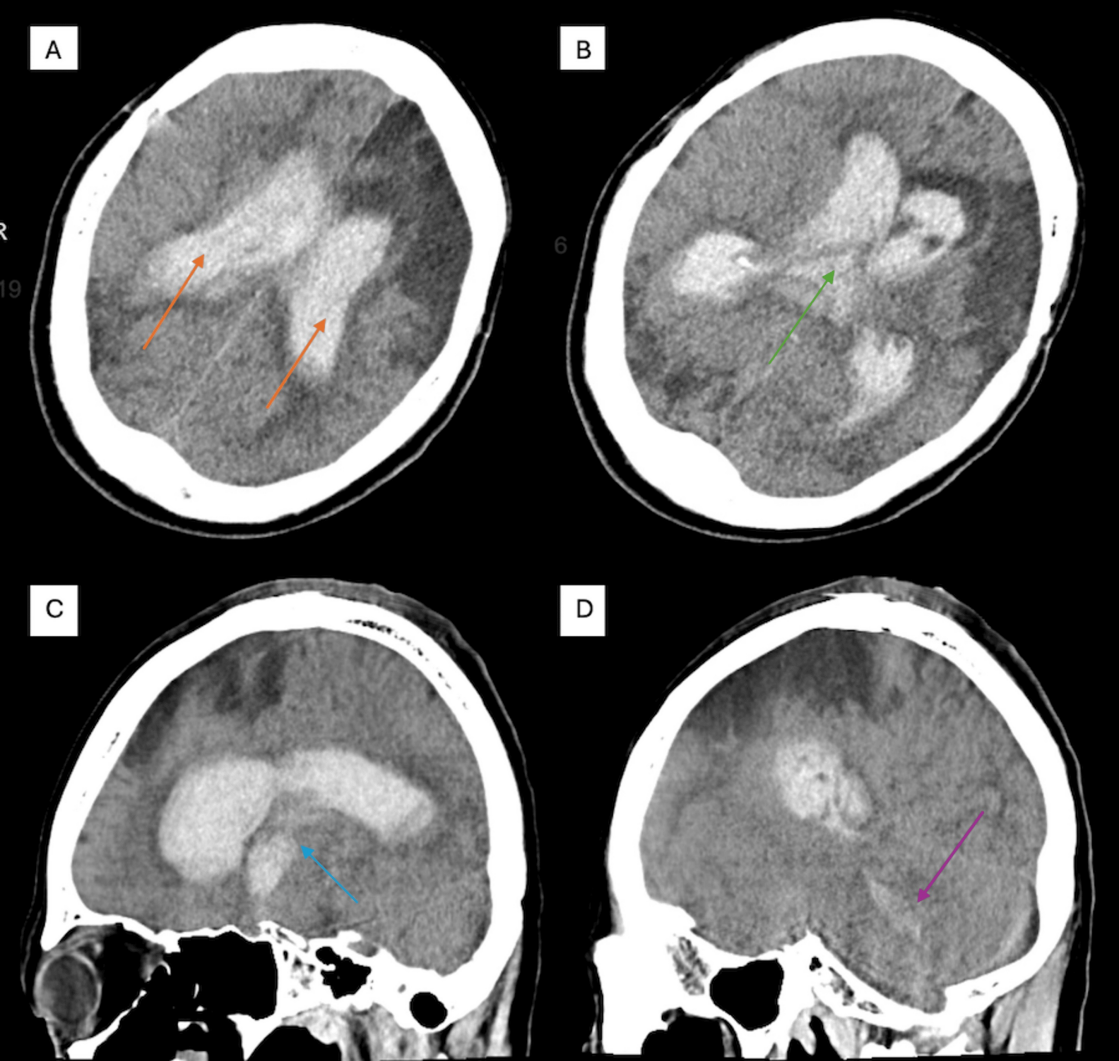
Initial CT head without contrast (A) large intraventricular hemorrhage occupying the bilateral lateral ventricles (orange arrows); (B-D) blood products in the third ventricle (green arrow), cerebral aqueduct (blue arrow), and cisterna magna (purple arrow) CT: computed tomography

Initial non-contrast head CT (Figure 1) demonstrated extensive IVH in the lateral and third ventricles, with blood extending into the cerebral aqueduct and cisterna magna, along with hydrocephalus and midline shift. The patient underwent emergent right-sided EVD placement.

**Figure 2:**
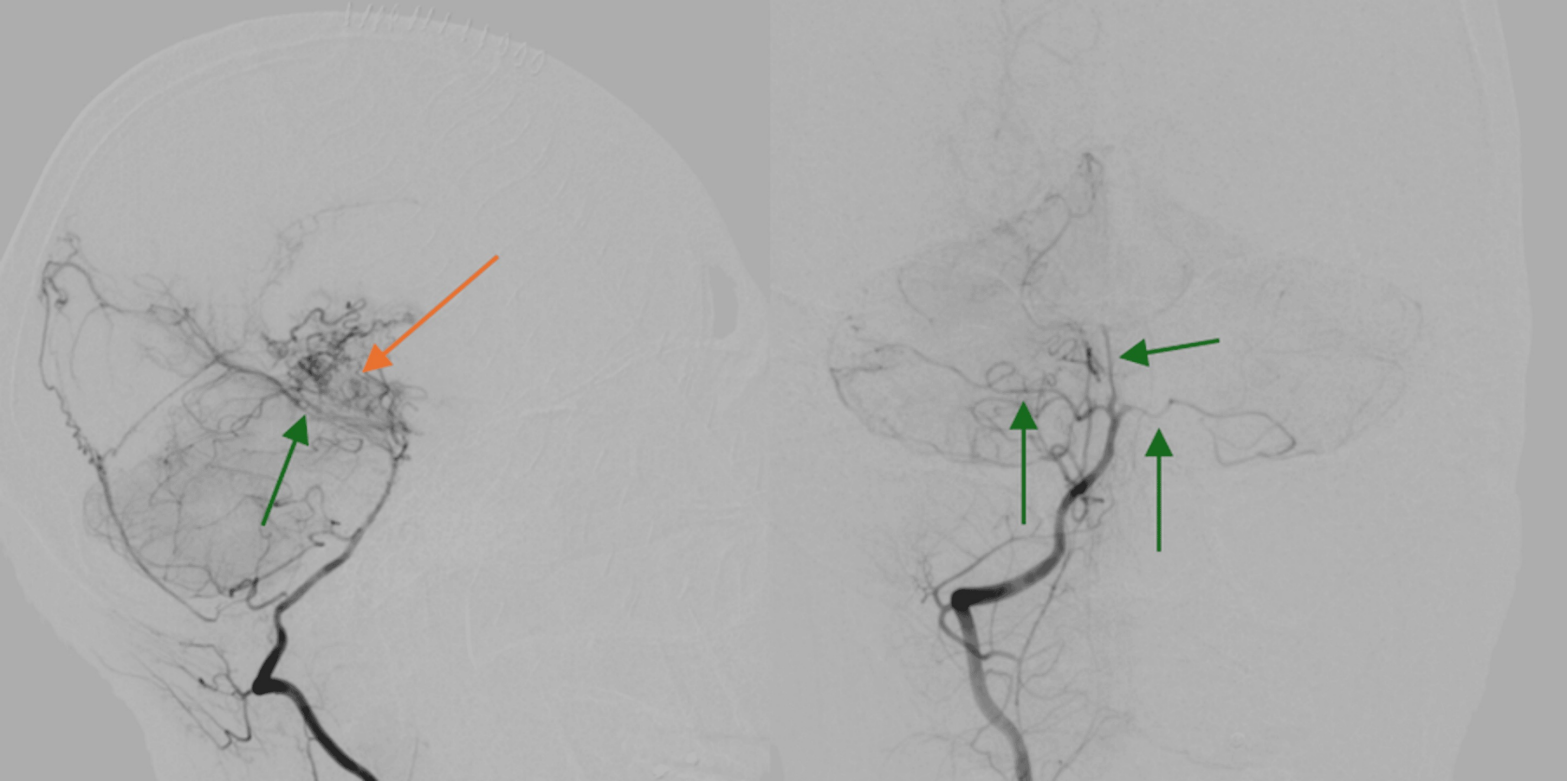
Cerebral angiogram Cerebral angiogram revealed severe stenosis of all major circumferential arteries, including the bilateral anterior cerebral arteries, middle cerebral arteries, and posterior cerebral arteries (green arrows). There was evidence of multiple tortuous vessels and fragile collaterals (orange arrow)

The patient remained in the surgical ICU (SICU) for six weeks. He developed aspiration pneumonia requiring broad-spectrum antibiotics and later a tracheostomy due to respiratory failure, and his neurological status remained poor. Cerebral angiography confirmed severe stenosis of all major intracranial vessels with classic "puff of smoke" collaterals (Figure 2).

Due to persistent IVH and worsening hydrocephalus despite initial EVD placement, the neurosurgical team proceeded with the placement of a VP shunt. Subsequent imaging confirmed accurate positioning, decreased ventricular size, and a significant reduction in intraventricular blood and hydrocephalus, indicating effective CSF drainage and decompression. Despite radiographic improvement, the patient remained tracheostomized, ventilator-dependent, and in a vegetative state. He was eventually transferred to a long-term care facility.

## Discussion

This case underscores the complex challenges of managing MMD in patients with CP, particularly in the setting of hemorrhagic complications and hydrocephalus. Although the patient underwent early bilateral EDAS to improve cerebral perfusion, he remained at risk for hemorrhagic stroke, highlighting the progressive and unpredictable nature of MMD despite surgical intervention. The development of IVH with secondary hydrocephalus further complicated his clinical course and ultimately contributed to poor neurological outcomes.

Diagnosing acute neurological deterioration in patients with CP is particularly challenging due to pre-existing motor deficits, cognitive impairments, and communication barriers. In this case, the patient’s nonverbal status and baseline functional limitations obscured early signs of decompensation. Seizure activity, a feature common to both CP and MMD, proved to be a critical signal prompting urgent neuroimaging. This illustrates the need for clinicians to maintain a high index of suspicion and to use surrogate markers, such as changes in seizure frequency, vital signs, or level of responsiveness, as potential indicators of acute pathology in non-communicative patients.

Improving outcomes in similar patients requires a proactive, multidisciplinary strategy. Lifelong surveillance with periodic imaging may help detect progressive vascular changes or new complications early. In high-risk individuals with CP and MMD, establishing individualized baseline neurological assessments, using caregiver reports to detect subtle deviations, and incorporating standardized seizure monitoring protocols can facilitate earlier intervention. Prompt recognition and treatment of complications like hydrocephalus or IVH are critical in preventing irreversible damage. Additionally, early involvement of neurology, neurosurgery, critical care, and rehabilitation teams can ensure comprehensive management across the continuum of care.

This report illustrates how the intersection of MMD and CP can obscure clinical deterioration and delay life-saving interventions. Optimizing surveillance and implementing tailored monitoring protocols are essential to improve functional outcomes and avoid devastating prognoses such as persistent vegetative state.

## Conclusions

This report underscores the complex interplay between MMD and CP, where baseline neurological deficits and impaired communication can delay recognition of acute decompensation. Despite early revascularization, our patient experienced catastrophic IVH and hydrocephalus, ultimately resulting in a persistent vegetative state. The report also highlights the pivotal role of neuroimaging - particularly CT, CTA, and DSA - in identifying acute complications such as IVH and hydrocephalus, assessing surgical outcomes, and confirming the diagnosis of MMD, especially when standard neurological assessment is limited. Clinicians must maintain a high index of suspicion and consider seizure activity or subtle behavioral changes as early indicators of cerebrovascular events in patients with comorbid neurodevelopmental disorders. Timely imaging, prompt management of hydrocephalus, and a multidisciplinary care model are essential to minimizing complications and improving long-term outcomes in this high-risk population.
